# Selection of Suitable Reference Genes for Normalization of Quantitative Real-Time Polymerase Chain Reaction in Human Cartilage Endplate of the Lumbar Spine

**DOI:** 10.1371/journal.pone.0088892

**Published:** 2014-02-18

**Authors:** Zhi-Jie Zhou, Jian-Feng Zhang, Ping Xia, Ji-Ying Wang, Shuai Chen, Xiang-Qian Fang, Shun-Wu Fan

**Affiliations:** 1 Department of Orthopaedic Surgery, Sir Run Run Shaw Hospital, School of Medicine, Zhejiang University, Hangzhou Zhejiang, China; 2 Department of Neurology, Sir Run Run Shaw Hospital, School of Medicine, Zhejiang University, Hangzhou, Zhejiang, China; 3 Sir Run Run Shaw Institute of Clinical Medicine of Zhejiang University, Hangzhou, Zhejiang, China; Northwestern University Feinberg School of Medicine, United States of America

## Abstract

The quantitative real-time polymerase chain reaction (qRT-PCR) is one of the most widely used methods to study gene expression profiles, and it requires appropriate normalization for accurate and reliable results. Although several genes are commonly used as reference genes (such as *GAPDH, ACTB,* and *18S rRNA*), they are also regulated and can be expressed at varying levels. In this study, we evaluated twelve well-known reference genes to identify the most suitable housekeeping gene for normalization of qRT-PCR in human lumbar vertebral endplate with Modic changes, by using the geNorm, NormFinder, and BestKeeper algorithms. Our results showed that the rarely-used *SDHA* was the most stable single reference gene, and a combination of three, *SDHA*, *B2M*, and *LDHA*, was the most suitable gene set for normalization in all samples. In addition, the commonly-used genes, *GAPDH*, *ACTB* and *18S rRNA*, were all inappropriate as internal standards. The rankings of reference genes for the three types of Modic change differed, although *SDHA* and *RPL13A* uniformly ranked in the first and last position, respectively. Further simulated expression analysis validated that the arbitrary use of a reference gene could lead to the misinterpretation of data. Our study confirmed the necessity of exploring the expression stability of potential reference genes in each specific tissue and experimental situation before quantitative evaluation of gene expression by qRT-PCR.

## Introduction

Signal intensity changes in the vertebral endplate and subchondral bone marrow on magnetic resonance imaging, also known as Modic changes (MCs), are often observed in patients with degenerative spinal diseases [1]. Three different types have been described. Type I changes are hypointense on T1-weighted imaging (T1WI) and hyperintense on T2-weighted imaging (T2WI) and indicate edema and hypervascularity in the lesions as confirmed histologically. Type II changes are hyperintense on T1WI and isointense or hyperintense on T2WI and reflect fatty replacement of the red bone marrow. Type III changes are hypointense on both T1WI and T2WI and represent subchondral bone sclerosis. MCs are strongly associated with intervertebral disc degeneration and low back pain [2]–[4]. Identification of the mechanisms and factors involved in the progression of MCs is of great importance for clinical interventions to repair or retard the development of MCs. To exploit the mechanisms of MCs, it is necessary to understand the pathophysiological changes of the vertebral cartilage endplate with MCs at the molecular level. Comprehensive evaluation of gene expression patterns is important for understanding the biological processes occurring in vertebral cartilage endplate with MCs.

Gene expression analysis is widely used in many fields of biological research [5], [6]. Gene expression analysis in cartilage has been satisfactorily performed in recent years since the major problem of poor ribonucleic acid (RNA) content in human cartilage is compensated by improving the RNA yield and quality and *via* cDNA amplification by *in vitro* transcription [7]–[10]. Quantitative real-time polymerase chain reaction (qRT-PCR) is currently one of the most precise and frequently-used methods to study the expression profiles of genes; it can quantify both the absolute and relative amounts of a gene’s RNA [11], [12]. Relative quantification is more common in qRT-PCR, and its accuracy, reliability, and reproducibility are highly dependent on the choice of suitable internal controls within each sample to normalize experimental variations [13]. The use of reference genes can correct biases caused by variations in RNA input, or reverse transcription efficiency, or amplification efficiency.

An ideal reference gene is presumed to be expressed at a constant level in all tissues and cells and under different experimental conditions. Although several genes are commonly used as controls (such as *GAPDH, ACTB,* and *18S rRNA*), they are also regulated and can be expressed at varying levels [11]. The use of inappropriate reference genes may weaken the detection sensitivity of the target genes and even lead to wrong results [14], [15]. Therefore, the expression stability of potential reference genes should be explored in each specific tissue and experimental situation. And the use of more than one reference gene is required for high-quality data, as suggested by many investigators [13], [16].

The suitability of reference genes in human osteoarthritic articular cartilage (hip and knee) has been evaluated by Pombo-Suarez et al. [17]. Their analysis showed that the expression levels of *GAPDH*, *ACTB*, and *18S rRNA* varied between samples. On the contrary, the rarely-used *TBP*, *RPL13A*, and *B2M* were the most stable and it was necessary to use several of these together to obtain the best results. So far, no appropriate reference genes have been identified in human lumbar cartilage endplate with MCs. In this study, we investigated the expression levels of 12 well-known reference genes to identify those most suitable for normalization of qRT-PCR in this tissue.

## Materials and Methods

### Human Lumbar Cartilage Endplate Sample Collection

Lumbar cartilage endplate specimens were obtained from patients who underwent lumbar interbody fusion for various spinal diseases in the Orthopaedic Surgery Department. All patients received a magnetic resonance imaging examination to determine the presence or absence of MCs in the lumbar vertebral endplate. Patients with MCs at the operated segment were recruited into the MC group, and those without MCs formed the control group. Finally, 12 MC and 12 non-MC samples, age- and sex-matched, were used. The Medical Ethics Committee of Sir Run Run Shaw Hospital, School of Medicine, Zhejiang University approved this study. All participants gave their written informed consent prior to participation. Patient characteristics are summarized in Table S1.

The cartilage endplate specimens were separated from disc and bone tissues immediately after they were harvested from the intervertebral space. They were then snap-frozen in liquid nitrogen and stored at −80°C until the extraction of total RNA.

### RNA Extraction Quality Control and cDNA Synthesis

RNA extraction from lumbar cartilage endplate was performed following the method of Untergasser [18]. Samples were first cut into small pieces under sterile conditions and ground in liquid nitrogen. Total RNA was then extracted using Trizol (Invitrogen) and further purified with an RNeasy Mini Kit (Qiagen) according to the manufacturer’s protocol with minor modifications. RNase-free DNase (Qiagen) was used twice to remove any trace of genomic DNA.

The concentration and purity of the isolated RNA were estimated in triplicate using a NanoDrop 2000 spectrophotometer (Thermo Scientific). Samples with concentrations ≥50 ng/µl and optical density absorption A_260_/A_280_ between 1.8 and 2.1 were taken for cDNA synthesis. The integrity of RNA samples was confirmed by electrophoresis on 2% Sybr Green agarose gel (Invitrogen) as indicated in the MIQE (*M*inimum *I*nformation for Publication of *Q*uantitative Real-Time PCR *E*xperiments) guidelines [19].

Total RNA (200 ng) was reversed-transcribed to first-strand cDNA using a Reverse Transcription System (Promega) in a total volume of 20 µl, according to the manufacturer’s instructions.

### Selection of Candidate Reference Genes and Primer Design

Twelve candidates were chosen based on their common use as reference genes and previous screening from microarray expression data of osteoarthritic cartilage [20], [21]. These genes represented several distinct functional classes so as to reduce the chances of co-regulation and false-positive reference gene selection.

For all genes, primer pairs were designed using Primer 3 ver. 0.4.0 (http://frodo.wi.mit.edu/primer3/) and then checked for the absence of stable hairpins and dimers using Oligo 5.0 (Molecular Biology Insights, Cascade, CO). All the primers were designed to be close to the 3′ end of the RNA sequence, and to be located on different exons to avoid genomic DNA contamination, except the primer for *18S rRNA* (Table 1). The primers were synthesized by Sangon Biotech Co., Ltd (Shanghai, China).

**Table 1:** Candidate reference genes and primer amplification efficiency.

Genesymbol	mRNA gene name	Genbankaccession No.	Primer sequence (F/R)	Ampliconsize (bp)	PCR efficiency(%)
IPO8	Importin 8	NM_006390.3	ccaaggggtggttcattctat	184	98.7
			tgtggtgggagaagcataatc		
RPL13A	Ribosomal protein L13A	NM_012423.2	gctgtgaaggcatcaacattt	245	95.1
			catccgctttttcttgtcgta		
TBP	TATA box binding protein	NM_003194.4	atgaggataagagagccacgaa	140	101.4
			gctggaaaacccaacttctgta		
SDHA	Succinate dehydrogenasecomplex, subunit A	NM_004168.2	agacctaaagcacctgaagacg	175	99.7
			atcaatccgcaccttgtagtct		
B2M	Beta-2-microglobulin	NM_004048.2	atccatccgacattgaagttg	150	98.5
			ggcaggcatactcatctttttc		
HPRT1	Hypoxanthine phosphoribosyl-transferase 1	NM_000194.2	cctggcgtcgtgattagtg	159	104.7
			tcccatctccttcatcacatc		
GUSB	Glucuronidase, beta	NM_000181.3	caatacctgactgacacctcca	205	97.8
			ggttactgcccttgacagagat		
LDHA	Lactate dehydrogenase A	NM_005566.3	gcctgtatggagtggaatgaa	157	100.6
			ccaggatgtgtagcctttgag		
HMBS	Hydroxymethylbilane synthase	NM_000190.3	gaaaacagcccaaagatgagag	238	103.4
			ggtccacttcattcttctccag		
ACTB	Actin, beta	NM_001101.3	agcgagcatcccccaaagtt	285	98.2
			gggcacgaaggctcatcatt		
GAPDH	Glyceraldehyde-3-phosphatedehydrogenase	NM_002046.3	agaaggctggggctcatttg	258	97.1
			aggggccatccacagtcttc		
18S	18S ribosomal RNA	NR_003286.2	cagccacccgagattgagca	253	96.9
			tagtagcgacgggcggtgtg		

### Quantitative Real-time PCR

qRT-PCR was performed using the GoTaq® qPCR Master Mix kit (Promega). Reactions were run in triplicate on a 7500 Real Time PCR System with 96-well plates (ABI). SYBR Green was used to detect dsDNA synthesis. Each reaction was performed in 20 µl containing 2 µl cDNA, 10 µl Gotaq Master Mix, 0.4 µl upstream and 0.4 µl downstream PCR primers (0.2 µM), and 7.2 µl nuclease-free water. Amplification was performed with an initial holding period at 95°C for 2 min, followed by a two-step PCR program consisting of 95°C for 5 s and 60°C for 34 s for 40 cycles. After 40 cycles, a melting analysis was performed by heating the amplicon from 60°C to 95°C. A reverse transcriptase negative control was included to ensure the absence of genomic DNA contamination, and the no-template control was also run to exclude contamination or dimer formation for each primer pair. The amplification specificity was confirmed by melting curve analysis and agarose gel electrophoresis of the products. For each primer pair, a series of 10-fold of three dilutions of cDNA (10- to 1,000-fold dilution) were made to generate a standard curve. The PCR amplification efficiency (E) was determined by the slope of the standard curve: E(%) = (10^[−1/slope]^ −1)×100%.

### Statistical Analysis

The Ct values for each sample were compiled and run through the Microsoft Excel-based software programs, geNorm (ver. 3.5) [16], NormFinder (Ver 0.953), [13] and BestKeeper [22]. These statistical algorithms were used to evaluate the stability of candidate reference genes, and then the overall ranking of the 12 candidate reference genes was determined according to the method described by Chen et al. [23].

For geNorm and NormFinder, data were analyzed by transforming raw Ct values into relative quantities using the ΔCt method. The lowest Ct value was subtracted from the raw Ct values of qRT-PCR for each gene to give the ΔCt value. The equation E^−ΔCt^ was applied to each data point. Therefore, all data were expressed relative to the expression of the most highly-expressed gene. BestKeeper analysis was based on the raw Ct values.

## Results

### Amplification Specificity and Efficiency and Expression Levels of 12 Candidate Reference Genes

Agarose gel electrophoresis and melting-curve analysis gave a single product of the expected length for each candidate gene. No non-specific amplicons or primer dimers were detected in the no-template control, and the absence of signals in the reverse transcriptase negative control suggested no genomic DNA contamination. All PCR assays showed efficiency values between 95.1% and 104.7% (Table 1).

**Figure 1:**
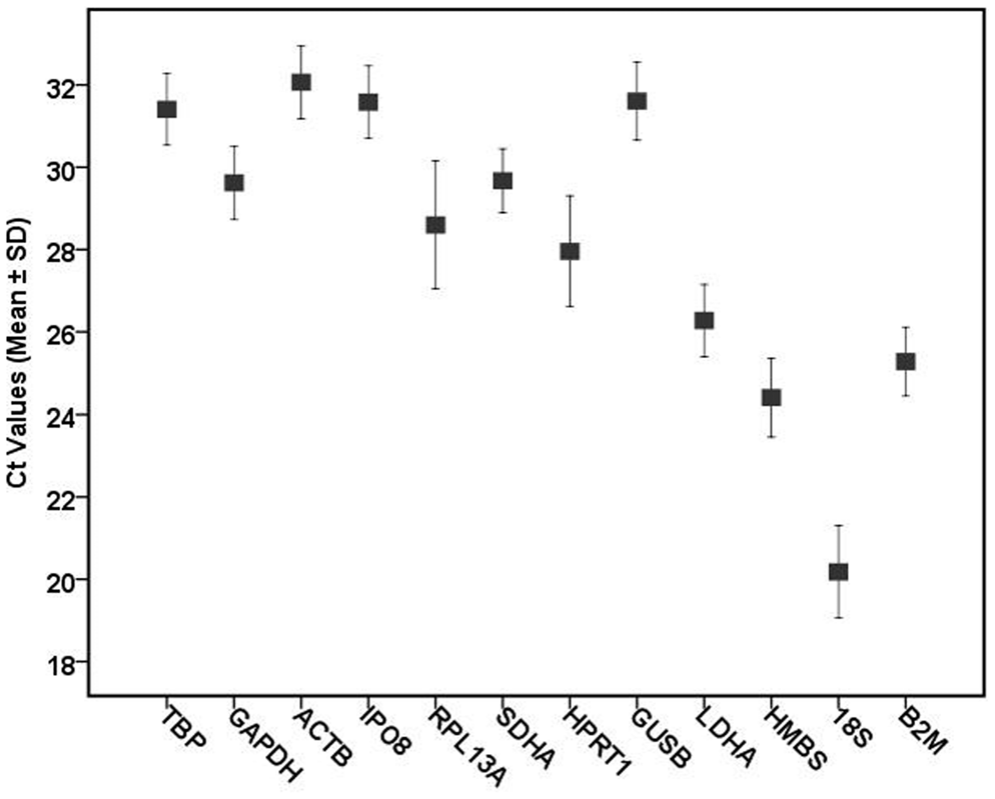
Expression levels of twelve candidate reference genes in different experimental sets. The boxes correspond to the mean Ct values; the upper and lower bars represent the standard deviation.

The expression levels of these 12 reference genes varied widely with Ct values ranging from 17.6 (*18S rRNA*) to 33.5 cycles (*ACTB*), and most of the Ct values were between 24 and 33 cycles. *18S rRNA* was the most abundantly transcribed with a mean Ct value of 20.2 cycles. *SDHA*, *GAPDH*, and *HRPT1* were moderately expressed with most of the Ct values between 26 and 31 cycles. *ACTB* showed the lowest level of expression with a mean Ct value as high as 32.1 cycles (Figure 1).

### Expression Stability of 12 Candidate Reference Genes

#### GeNorm analysis

**Figure 2:**
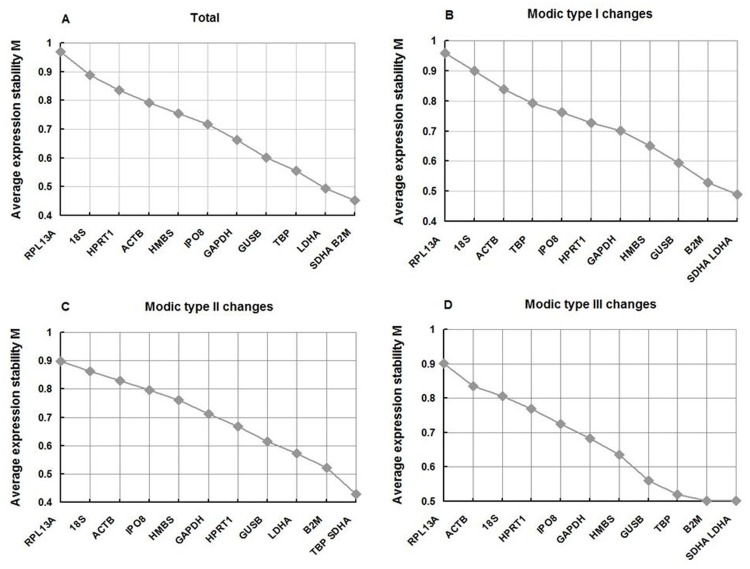
Gene expression stability and ranking of 12 candidate reference genes as calculated by geNorm. Average expression stability values (M) following stepwise exclusion of the least-stable gene across all samples and different subsets. A lower M value indicates more stable expression. Samples of (a) total cartilage endplate, (b) MC type I, (c) MC type II, and (d) MC type III.

GeNorm is a program designed to analyze the expression stability of candidate reference genes on the assumption that the ratio of the expression level of two ideal reference genes is constant in all samples. And the average expression-stability M value for each investigated gene is calculated with the average of pairwise variations, according to which the expression stability of all reference genes is ranked. Genes with the lowest M value are the most stable, and a value of 1.5 is recommended as the cut-off for the selection of qRT-PCR reference genes by Vandesompele et al. [16]. In our analysis, when the results from all 24 samples of cartilage endplate were combined, the genes with the smallest M value were *SDHA* and *B2M* (0.45), therefore these were the most stable genes. The sequence from most to least stable was *SDHA, B2M, LDHA*, *TBP*, *GUSB*, *GAPDH*, *IPO8, HMBS*, *ACTB*, *HPRT1*, *18S rRNA*, and *RPL13A*, with the largest M value being 0.97 for *RPL13A* (Figure 2A). In subgroup analysis, *SDHA* and *LDHA*, *SDHA* and *TBP*, and *SDHA* and *LDHA* were the most stable genes for MC types I, II, and III, respectively, and *RPL13A* was uniformly the most unstable (Figure 2B–2D). The M values of the 12 genes in all samples and subsets were less than 1, below the default limit of M ≤1.5, indicating relatively high expression stability (Figure 2).

**Figure 3:**
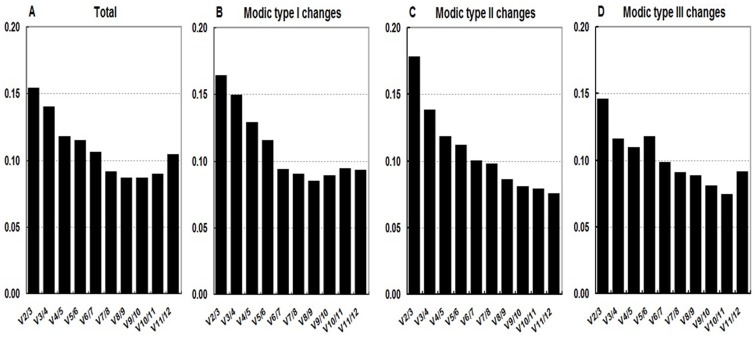
Determination of optimal number of reference genes for each subset according to geNorm. The pairwise variation value (V_n_/V_n+1_) reflects the improvement obtained by the inclusion of an additional reference gene and was used to determine the optimal number of reference genes. Samples of (a) total cartilage endplate, (b) MC type I, (c) MC type II, and (d) MC type III.

The geNorm algorithm also calculates the pairwise variation (V_n_/V_n+1_) between two sequential normalization factors NF_n_ and NF_n+1_ to determine the optimum number of reference genes. As a general rule, the stepwise inclusion of reference genes is performed until V_n_/V_n+1_ drops below the theoretical threshold of 0.15, when the benefit of adding an extra gene (n+1) is limited for accuracy normalization [17], [24]. And the use of at least the three most stable reference genes is recommended [16]. In this study, the pairwise variations V_3/4_ for total and subgroup analyses were all below 0.15, therefore the addition of the fourth-best gene to the gene set composed of the three best ones was not needed (Figure 3).

#### NormFinder analysis

NormFinder is a model-based algorithm, which identifies the most stable reference genes based on combing samples into groups. The main goal of this approach is to calculate the inter- and intra-group variation of the candidate reference genes and then combine both results into a stability value M. Genes with the lowest value are considered to be the most stable [13].

The calculated stability values of the 12 genes are listed in Table 2. According to NormFinder, the most stable reference gene in all samples was *LDHA*, with a M value of 0.102, followed by *SDHA*, *B2M*, *GUSB*, *GAPDH*, *IPO8*, *HMBS*, *ACTB*, *TBP*, *18S rRNA*, *HPRT1*, and *RPL13A*. *LDHA* was also top-ranked for those with MC types I and III, while *B2M* was the most stable for those with MC type II. *SDHA* was among the three most stable genes and *RPL13A* was the most unstable in the total sample set as well as in the three subsets, as for the geNorm algorithm.

**Table 2:** Ranking of candidate reference genes in order of their expression stability as calculated by NormFinder.

Rank	Total	Modic type I changes	Modic type II changes	Modic type III changes
1	LDHA	LDHA	B2M	LDHA
M value	0.102	0.133	0.177	0.107
2	SDHA	SDHA	SDHA	B2M
M value	0.108	0.147	0.187	0.176
3	B2M	IPO8	GUSB	SDHA
M value	0.111	0.242	0.192	0.180
4	GUSB	B2M	LDHA	GUSB
M value	0.135	0.296	0.210	0.197
5	GAPDH	HMBS	GAPDH	HMBS
M value	0.146	0.335	0.212	0.227
6	IPO8	GAPDH	HPRT1	18S
M value	0.157	0.344	0.228	0.256
7	HMBS	GUSB	ACTB	GAPDH
M value	0.157	0.364	0.229	0.281
8	ACTB	ACTB	IPO8	ACTB
M value	0.163	0.405	0.236	0.283
9	TBP	TBP	18S	IPO8
M value	0.173	0.435	0.289	0.287
10	18S	18S	HMBS	TBP
M value	0.193	0.609	0.295	0.299
11	HPRT1	HPRT1	TBP	HPRT1
M value	0.199	0.634	0.304	0.518
12	RPL13A	RPL13A	RPL13A	RPL13A
M value	0.249	0.730	0.320	0.603

#### BestKeeper analysis

BestKeeper analyzes the expression stability of reference genes using raw Ct values. Gene expression variation is determined by the calculated standard deviation (SD) and coefficient of variance (CV) for all candidate reference genes based on the whole data set of their Ct values [22]. Those with the lowest CV±SD were identified as the most stable genes. Those with SD values >1 were considered to be inconsistent and were excluded. In the current analysis, *SDHA* and *ACTB* had the smallest CV ± SD of 2.614±0.776 and 2.757±0.884, respectively, thus being the most stable reference genes in all samples. This was different from the results produced by geNorm and NormFinder, in which *ACTB* ranked poorly (8/12 and 9/12, respectively). In the three subsets, *SDHA* was the most stably expressed and *18r RNA* or *RPL13A* the least stably expressed (Table 3).

**Table 3:** Ranking of candidate reference genes in order of their expression stability as calculated by BestKeeper.

Rank	Total	Modic type I changes	Modic type II changes	Modic type III changes
1	SDHA	SDHA	SDHA	SDHA
CV±SD	2.614±0.776	2.441±0.720	2.593±0.765	2.928±0.869
2	ACTB	LDHA	TBP	TBP
CV±SD	2.757±0.884	2.497±0.646	2.714±0.851	2.955±0.934
3	TBP	IPO8	ACTB	IPO8
CV±SD	2.770±0.870	2.745±0.860	2.887±0.924	2.974±0.935
4	IPO8	GUSB	GUSB	GUSB
CV±SD	2.797±0.883	2.760±0.866	3.058±0.959	3.017±0.949
5	GUSB	TBP	IPO8	ACTB
CV±SD	3.013±0.952	2.762±0.863	3.127±0.985	3.114±0.996
6	GAPDH	ACTB	B2M	LDHA
CV±SD	3.019±0.894	2.868±0.910	3.291±0.824	3.415±0.892
7	B2M	HMBS	GAPDH	GAPDH
CV±SD	3.318±0.839	3.030±0.735	3.299±0.972	3.517±1.032
8	LDHA	B2M	LDHA	B2M
CV±SD	3.329±0.875	3.129±0.783	3.380±0.884	3.687±0.928
9	HMBS	GAPDH	HPRT1	HMBS
CV±SD	3.899±0.952	3.538±0.910	3.611±0.990	3.979±0.971
10	HPRT1	HPRT1	HMBS	18S
CV±SD	4.827±1.349	3.782±1.045	3.672±0.887	4.938±0.994
11	RPL13A	RPL13A	RPL13A	HPRT1
CV±SD	5.435±1.554	3.964±1.104	4.039±1.154	5.054±1.408
12	18S	18S	18S	RPL13A
CV±SD	5.553±1.121	4.201±0.822	4.505±0.907	5.366±1.536

#### Final ranking of candidate reference genes

Since the discrepancies in expression stability of candidate reference genes among the algorithms, a method taking into account all the three sets of results was applied to calculate the final ranking. Specifically, the geometric means of the three ranking numbers produced by geNorm, NormFinder, and BestKeeper were calculated for each candidate reference gene; those with the smallest geometric means were considered to be the most stable [23]. As a result, *SDHA* was the most stable single gene in all samples, and *SDHA*, *B2M*, and *LDHA* comprised the optimal reference gene set. Although the rankings of reference genes for the three types of MC were different, *SDHA* and *RPL13A* uniformly ranked in the first and last position, respectively (Table 4).

**Table 4:** Overall ranking of twelve candidate reference genes.

Rank	Total	Modic type I changes	Modic type II changes	Modic type III changes
1	SDHA	SDHA	SDHA	SDHA
geometric mean	1.26	1.26	1.26	1.44
2	B2M	LDHA	B2M	LDHA
geometric mean	2.76	1.26	2.62	1.82
3	LDHA	IPO8	TBP	B2M
geometric mean	2.88	4.16	2.80	3.63
4	GUSB	B2M	GUSB	TBP
geometric mean	4.64	4.58	3.91	4.31
5	TBP	GUSB	LDHA	GUSB
geometric mean	4.76	4.82	5.04	4.31
6	ACTB	HMBS	ACTB	IPO8
geometric mean	5.24	5.59	5.94	6.00
7	IPO8	GAPDH	GAPDH	HMBS
geometric mean	5.52	6.87	6.26	6.46
8	GAPDH	TBP	HPRT1	GAPDH
geometric mean	5.65	7.40	6.87	7.00
9	HMBS	ACTB	IPO8	ACTB
geometric mean	7.96	7.83	7.11	7.61
10	HPRT1	HPRT1	HMBS	18S
geometric mean	10.32	9.17	9.28	8.43
11	18S	18S	18S	HPRT1
geometric mean	10.97	10.97	10.59	10.29
12	RPL13A	RPL13A	RPL13A	RPL13A
geometric mean	11.66	11.66	11.66	12.00

### Effect of Choice of Reference Genes

**Figure 4:**
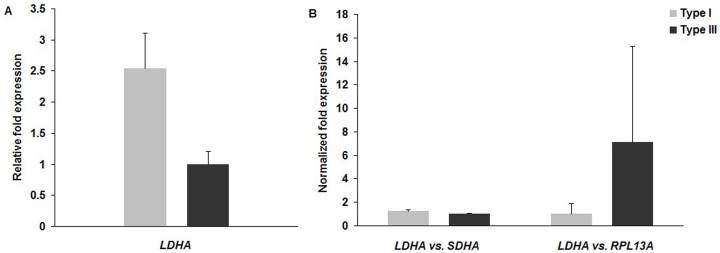
Effect of choice of reference genes. Relative (a) and normalized (b) fold expression of *LDHA*. Simulated expression analysis was performed using data from samples with MC types I and III, and *SDHA* and *RPL13A* were set as calibrators.

To validate the importance of selecting the appropriate reference genes for normalization, a simulated expression analysis was performed using data from samples with MC types I and III. In these two types of specimen, *SDHA* and *LDHA* consistently turned out to be the most stable genes, and *RPL13A* the most unstable. The simulation was conducted taking *LDHA* as target and *SDHA* and *RPL13A* as reference genes (Figure 4). The relative fold expression of *LDHA* was calculated and normalized to the lowest value: 2.54±0.57 in samples with MC type I and 1.00±0.21 in those with MC type III; the different between these two subgroups was significant (P  = 0.012). When the expression level of *LDHA* was normalized to *SDHA*, it was higher in the subgroup with MC type I than that with type III (1.27±0.08 *versus* 1.00±0.06, P  = 0.009). However, *LDHA* was non-significantly less expressed in the former than the latter group when using *RPL13A* as the reference (1.00±0.89 *versus* 7.09±8.20, P  = 0.219) (Figure 4). Therefore, the difference of gene expression levels between the subgroups could be masked by using unsuitable reference genes.

## Discussion

The purpose of this study was to choose appropriate internal controls to ensure credible evaluation of gene expression levels in human lumbar cartilage endplate with MCs. All the 12 candidates were selected from previous investigations, and they were reported to have relatively stable expression levels [13], [16], [17], [25]. Our studies showed that the most stable gene in all samples was *SDHA*, which is among the less commonly used. And a combination of three reference genes was recommended, i.e., *SDHA*, *B2M*, and *LDHA*, based on a comprehensive consideration of the results of all algorithms. The application of multiple references is beneficial for normalization [13], [17], [26].

We compared our results with those of a trial which selected reference genes for normalization in human osteoarthritic hip and knee cartilage [17]. *B2M* was identified as one of the best reference genes in both studies. The surprising result in this analysis was that *GAPDH* as well as *ACTB* and *18S rRNA* performed poorly, and this has also been reported for osteoarthritic articular cartilage [17]. These genes are often given preference in many studies [16], [27]. Our results, however, further confirmed the necessity of assessing the reference genes in each tissue and experimental condition. Yet there were discrepancies between our results and the published data for hip and knee cartilage. They concluded that *RPL13A* is among the most stable reference genes, while *SDHA* is not. On the contrary, we found that *SDHA* was the most stable while *RPL13A* the most unstable. Thus, in tissues from different anatomical regions, hip and knee joint cartilage *versus* lumbar vertebral endplate cartilage, the stability of reference genes is different.

Interestingly, the results for the three types of MC differed. For instance, *IPO8*, which was one of the most stably-expressed genes in samples with MC type I, was not ranked so high in MC types II and III. Similarly, *TBP* was relatively stable in samples from MC types II and III, however, it was ranked lower in MC type I. Therefore, even for a tissue with different subtypes of pathologic change, previous testing of the stability of reference genes is required.

The geNorm, NormFinder, and BestKeeper are now widely used for the selection of stable reference genes with invariable expression from a set of candidate genes. GeNorm analyzes the expression stability of the tested genes in all samples, and ranks them according to a stability measure [16]. In contrast, NormFinder evaluates the expression stability of each single reference gene independently, and takes into account intra- and inter-group variations for normalization [13]. While BestKeeper analyzes the stability of candidate reference genes depending on the standard deviation and coefficient of variance of their Ct values. Together they seek genes with stable expression levels, by either the relative stability with reference to other candidates, or to clinically-relevant groups, or to their intrinsic degree of variation [13], [16], [22]. The use of these programs provides complementary information. Because of the distinct statistical algorithms used by these three programs, it was not surprising that they gave somewhat different results. Nevertheless, there was general agreement; *SDHA* was in the top two positions and *RPL13A* in the last two in all samples and subsets for these three programs.

There were some limitations in our study. First, we only included a limited number of candidate genes, and it is likely that some other genes may be better internal references for human lumbar endplate cartilage with MCs. Second, our results only apply directly to vertebral cartilage endplate with MCs in the lumbar region. It is unclear how well our results could be extended to other regions of vertebral cartilage endplate, i.e., the cervical and thoracic regions.

## Supporting Information

Table S1 **Characteristics of controls and patients with Modic changes.**
(DOC)
