# Supplementary material for: Selection of Suitable Reference Genes for Normalization of Quantitative Real-Time Polymerase Chain Reaction in Human Cartilage Endplate of the Lumbar Spine
Source: PLoS One. 2014 Feb 18;9(2):e88892. doi: 10.1371/journal.pone.0088892 (PMC3928306; doi:10.1371/journal.pone.0088892)
Supplement: Table S1 — Characteristics of controls and patients with Modic changes. (DOC) [file pone.0088892.s001.doc]

**Table S1.** Characteristics of the controls and patients with Modic changes.

| **Case No.** | **Sex** | **Age**  **(yrs)** | **Diagnosis** | **Modic**  **changes** | **Lumbar segment** |
| --- | --- | --- | --- | --- | --- |
| C2098761 | M | 31 | spinal fracture | None | L_4-5_ |
| C1876384 | M | 28 | spinal fracture | None | L_2-3_ |
| C2146652 | F | 24 | spinal fracture | None | L_1-2_ |
| C1786760 | F | 56 | spinal fracture | None | L_4-5_ |
| C1897764 | M | 69 | spinal tumor | None | L_5-_S_1_ |
| C1897652 | F | 47 | spinal tumor | None | L_3-4_ |
| C1786653 | M | 24 | spinal tuberculosis | None | L_5-_S_1_ |
| C1786534 | M | 53 | spinal tuberculosis | None | L_3-4_ |
| C2487876 | M | 46 | degenerative spondylolisthesis | None | L_5-_S_1_ |
| C2166358 | F | 61 | degenerative disc disease | None | L_5-_S_1_ |
| C1896734 | M | 49 | disc herniation | None | L_4-5_ |
| C2174256 | F | 56 | spinal stenosis | None | L_4-5_ |
| M2068354 | M | 53 | degenerative spondylolisthesis | Type I | L_4-5_ |
| M1956137 | M | 39 | degenerative disc disease | Type I | L_2-3_ |
| M2197748 | F | 23 | isthmic spondylolisthesis | Type I | L_4-5_ |
| M1936654 | M | 61 | degenerative spondylolisthesis | Type II | L_5-_S_1_ |
| M1876349 | M | 65 | degenerative spondylolisthesis | Type II | L_4-5_ |
| M1942675 | M | 32 | degenerative disc disease | Type II | L_3-4_ |
| M1886755 | F | 45 | degenerative disc disease | Type II | L_4-5_ |
| M2176393 | F | 58 | degenerative disc disease | Type II | L_4-5_ |
| M1965327 | M | 44 | spinal stenosis | Type II | L_5-_S_1_ |
| M2071272 | F | 48 | degenerative disc disease | Type III | L_3-4_ |
| M1986254 | M | 52 | degenerative spondylolisthesis | Type III | L_5-_S_1_ |
| M1755639 | M | 43 | spinal stenosis | Type III | L_4-5_ |

F, female; M, male; L, lumbar;
